# Internal Carotid Artery Stenosis Presenting with Limb Shaking TIA

**DOI:** 10.1155/2016/3656859

**Published:** 2016-10-20

**Authors:** Awad Javaid, Mostafa Alfishawy

**Affiliations:** Internal Medicine Department, Queens Hospital Center, 82-68 164th St., Jamaica, NY 11432, USA

## Abstract

Internal carotid artery (ICA) stenosis may lead to a wide range of clinical symptoms. We describe the case of a 66-year-old female who experienced a transient ischemic attack (TIA) with episodes of limb shaking caused by ICA stenosis. After epilepsy had been suspected and ruled out, studies of her left ICA showed extensive blockage as a result of atherosclerosis. Magnetic resonance angiography (MRA) revealed total occlusion of the left ICA and the patient was eventually medically managed due to the strong possibility of surgical complications. We reported this patient's clinical course to shed light on a rare manifestation of carotid stenosis that may be confused with other diagnoses if not closely scrutinized.

## 1. Introduction

Patients with ICA stenosis typically present with TIA, stroke, or no symptoms at all depending on the degree of atherosclerosis. TIA often causes focal neurologic deficits such as limb weakness, vision loss, or aphasia. Infrequently, some patients with severe carotid stenosis may experience a TIA accompanied by limb shaking, which can be confused with a seizure.

## 2. Case Presentation

A 66-year-old female visiting the United States from Bangladesh with a past medical history of hypertension, type II diabetes mellitus, and hypothyroidism checked into the emergency department after experiencing two episodes of jerking and shivering. Her daughter witnessed the events and described them as shaking and shivering of her mother's arms and neck lasting less than two minutes. The patient was lying in bed with her head turned to the left and complained of neck pain and dizziness when the shaking began. She did not lose consciousness, bite her tongue, urinate, or experience any common postictal symptoms. The first episode occurred at 4 am and the second episode took place at 1 pm. She had experienced these symptoms three to four times per year for the last two to three years. Separately, the patient had also been intermittently experiencing a sensation of the room spinning over the past three days that was causing her to lose her balance. The patient denied syncope, fall, dysuria, abdominal pain, and back pain. Her vital signs in the emergency department were as follows: blood pressure 177/71 mmHg, heart rate 57 bpm, respiration rate 16/min, temperature 98.3°F, and peripheral capillary oxygen saturation 96% on room air. The patient was oriented to person, place, and time. Her physical exam was positive for a right carotid bruit and decreased sensation to pinprick, soft touch, and vibration in the left lower extremity. Otherwise, the patient's cranial nerves II-XII were grossly intact, and she presented with no weakness or focal neurological deficit.

**Figure 1:**
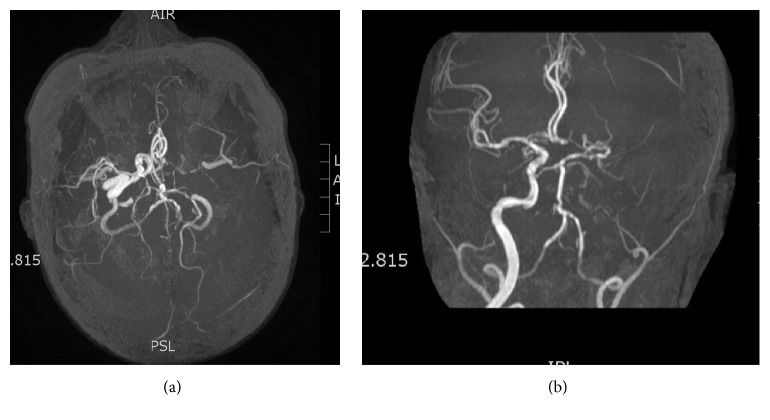
Magnetic resonance angiography showing markedly reduced flow in the left ICA.

Emergency department physicians administered meclizine for the patient's dizziness and performed a chest X-ray, which revealed no irregularities. Electroencephalogram (EEG) and head computerized tomography (CT) failed to show any abnormalities. Cerebral MRA revealed a lack of flow along the course of the intracranial left internal carotid artery extending to the supraclinoid segment, with collateral circulation noted (Figure 1). Therefore, a decision was made to admit the patient to the general medicine inpatient unit in light of her symptoms and MRA result. Although normal EEG between ischemic episodes cannot rule out epilepsy, the results of the MRA and lack of classic epileptic symptoms led neurologists not to pursue further neurophysiological testing. The patient was given aspirin, Norvasc, losartan, Tenormin, Robaxin, metformin, and insulin. Meclizine was continued, and neurologists recommended adding Plavix to her treatment. She was also given Lovenox prophylaxis for deep vein thrombosis. Vascular surgeons suggested carotid duplex study, which exhibited complete occlusion of the left ICA and 20% to 30% occlusion of the right ICA. Vascular surgeons decided not to pursue surgical intervention for the left ICA due to the risks associated with the procedure. Over the course of a six-day hospital stay, the patient did not experience any further episodes of limb shaking; therefore she was discharged with the approval of neurologists and cardiologists. The final diagnosis of the patient's condition was TIA with limb shaking.

## 3. Discussion

Atherosclerosis is characterized by the presence of intimal lesions, which are referred to as atherosclerotic or atheromatous plaques [1]. These are raised lesions of soft lipid cores composed mainly of cholesterol and cholesterol esters with necrotic debris. Plaques are covered by fibrous caps and can mechanically obstruct the vascular lumen. They are prone to rupture and can result in catastrophic vessel thrombosis. Plaques also weaken the intima media, which can sometimes lead to aneurysm formation [1]. At early stages, remodeling of the media preserves the luminal diameter by increasing the vessel circumference. Due to limits on remodeling, eventually, the expanding atheroma impinges on blood flow and causes stenosis [1]. Critical stenosis occurs approximately at 70% fixed occlusion and limits flow so severely that tissue demand exceeds perfusion [1].

Patients with carotid atherosclerosis usually present with a carotid bruit, ischemic symptoms, or stroke. TIAs may be due to either low flow or embolization. When TIAs are due to reduced flow with inadequate collateral blood supply, they are brief, repetitive, stereotyped spells [2]. Embolic TIAs are usually single and more prolonged, and the symptoms are related to the vascular territories involved [3]. Ocular ischemia can cause blindness and absent pupillary light response [3]. Funduscopic examination may demonstrate arterial occlusion or ischemic damage to the retina [3]. Ischemia to the brain from carotid disease may cause contralateral homonymous hemianopsia, hemiparesis, or hemisensory loss. Left hemisphere ischemia may cause aphasia, while right hemisphere ischemia may cause visuospatial neglect, apraxia (inability to perform purposeful movements), and dysprosody (variations in melody, intonation, pauses, stresses, intensity, or accents of speech) [4]. Atypical symptoms of internal carotid artery stenosis include limb shaking and unilateral transient loss of vision after exposure to bright light or syncope [5].

Limb shaking is an unusual presentation of ICA stenosis which can pose a diagnostic dilemma due to similar presentation to epilepsy. Limb shaking as a manifestation of TIA was first described in 1962 by Fisher [6]. It is difficult to locate case reports of these episodes in patients living in Bangladesh and the Indian subcontinent as a whole, but at least two cases were described in India and one was described in Pakistan [7–9]. Limb shaking is caused by transient hypoperfusion at vessels which can no longer dilate or constrict due to atherosclerosis. Usually, the patient experiences transient rhythmic or arrhythmic involuntary movements [10]. Facial muscles are always spared, and EEG reveals the absence of abnormalities [11]. Limb shaking TIA can be differentiated from seizure based on the absence of aura, loss of consciousness, incontinence, and tongue biting. Additionally, anticonvulsant medications fail to improve symptoms. Another important feature apparent in some patients with limb shaking TIA is the onset of symptoms after actions which cause cerebral hypoperfusion, such as rising from seated position [12]. Transient ischemia in the patient described in this report most likely occurred due to prolonged rotation of her head to the left side while lying down, which caused ischemia of the right sided, intact arteries.

The American Heart Association and American Stroke Association presented guidelines for prevention of stroke and management of patients with extracranial carotid and vertebral artery disease in 2014 and 2011. Treatment options for patients with symptomatic carotid atherosclerotic disease include carotid endarterectomy (CEA), carotid artery stenting (CAS), and medical management. Patients with recently symptomatic carotid stenosis of 70 to 99 percent who have a life expectancy of at least five years are recommended to undergo CEA [13–15]. Generally, CEA is preferred because randomized trials have demonstrated that periprocedural (30 days) stroke or death rate is greater with stenting than with endarterectomy [16]. CAS is preferred for patients who have a carotid lesion that is not suitable for surgical access, who have radiation-induced stenosis, or who have clinically significant cardiac, pulmonary, or other disease that greatly increases the risk of anesthesia and surgical operation [17]. Medical management is preferred for symptomatic carotid stenosis that is less than 50 percent [17]. Medically stable asymptomatic patients who have a life expectancy of at least five years with 80% or greater carotid stenosis are also recommended to get CEA.

Endarterectomy is a viable option only when residual flow is present. If there is total occlusion, medical management is the only realistic option. Multiple studies have failed to show clinical benefit of revascularization in patients with complete occlusion, compared to medical management alone [18, 19]. Medical management consists of aspirin, statins, antiplatelet agents, antihypertensive agents, and lifestyle modification consisting of smoking cessation, limited alcohol consumption, weight loss, regular exercise, and a well-balanced diet. The decision was made to medically manage the patient in this case on account of her being a poor surgical candidate due to complete occlusion of the left ICA and limited occlusion of the right ICA. Currently, the patient's hypertension, diabetes, and hypothyroidism are being monitored at periodic clinic visits.

## 4. Conclusion

Physicians should be careful to differentiate limb shaking caused by ICA from a seizure episode. A timely diagnosis of limb shaking due to ICA is crucial in that it can prevent possible complications of a TIA or stroke, as well as the administration of unnecessary medication regimens for epilepsy.
